# The Molecular and Structural Characterization of Two Vitellogenins from the Free-Living Nematode *Oscheius tipulae*

**DOI:** 10.1371/journal.pone.0053460

**Published:** 2013-01-07

**Authors:** Daniela P. Almenara, Joselene P. de Moura, Cristiane P. Scarabotto, Russolina B. Zingali, Carlos E. Winter

**Affiliations:** 1 Department of Parasitology, Institute of Biomedical Sciences – University of São Paulo, São Paulo, Brazil; 2 Laboratory of Proteomics and Protein and Peptide Microsequencing, Institute of Medical Biochemistry - UFRJ/CCS/Bloco H, Cid. Universitária – Ilha do Fundão, Rio de Janeiro, Brazil; The University of Chicago, United States of America

## Abstract

This paper describes the purification of yolk proteins, which are important for the reproduction of egg-laying animals, and the structural characterization of two vitellogenins, VT1 and OTI-VIT-6, of the nematode *Oscheius tipulae*. *O. tipulae* is an alternative model organism to its relative, the widely used *Caenorhabditis elegans*, and is a good model to understand reproduction in insect parasitic nematodes of the genus *Heterorhabditis*. The native purified *O. tipulae* vitellogenin is composed of three polypeptides (VT1, VT2 and VT3), whereas in *C. elegans*, vitellogenin is composed of four polypeptides. The gene (*Oti-vit-1*) encoding yolk polypeptide VT1 has been recently identified in the genome of *O. tipulae*. Immunoblotting and N-terminal sequencing confirmed that VT1 is indeed coded by *Oti-vit-1*. Utilizing the same experimental approaches, we showed that the polypeptides VT2 and VT3 are derived from the proteolytic processing of the C- and N-terminal portions of the precursor OTI-VIT-6, respectively. We also showed that the recombinant polypeptide (P40), corresponding to the N-terminal sequence of OTI-VIT-6, preferentially interacts with a 100-kDa polypeptide found in adult worm extracts, as we have previously shown for the native vitellins of *O. tipulae*. Using the putative nematode vitellogenin amino acid sequences available in the UniProtKB database, we constructed a phylogenetic tree and showed that the *O. tipulae* vitellogenins characterized in this study are orthologous to those of the *Caenorhabditis* spp. Together, these results represent the first structural and functional comparative study of nematode yolk proteins outside the *Caenorhabditis* genus and provide insight into the evolution of these lipoproteins within the Nematode Phylum.

## Introduction

Nematode egg production is a key strategy for the colonization of new habitats and hosts. Egg-laying animals deposit proteins, lipids, carbohydrates and other yolk reserves in their eggs that are used by the embryo during its development [1]. The primary stored proteins are the vitellins, which are lipoproteins that share a common ancestor that predates the origin of the bilateral metazoans [1], [2], [3], [4], [5], [6]. Vitellins originate from an extra-ovarian protein, named vitellogenin, utilized by the growing oocyte [7]. The uptake of vitellogenin occurs through membrane receptors present in the oocyte cell membrane [8], [9]. Once inside the oocytes, vitellogenin is processed in endosomal compartments and stored in the yolk granules until further use by the developing embryo or recently emerged L1 larvae [10]. Traditionally, yolk proteins and vitellogenins have been studied to understand the structure and function of other lipoproteins, including human apolipoprotein B [11] or in environmental research [12].

One to six different genes encode vitellogenins [13], [14], [15]. Several of these genes have been isolated and studied in nematodes [16], [5], insects [17], amphibians [18], birds [19], several fishes [20], [21], [22] and cyclostoms [23], among other species.

Free-living nematodes represent excellent model animals for molecular studies of the different aspects of vitellogenesis. The large amount of eggs produced by parasitic nematodes, such as *Ascaris* [24], and the short life cycle of the free-living species make these worms the best animal models in which to study several aspects of vitellogenesis. The development and reproductive behavior of the free-living species *Caenorhabditis elegans* has been extensively studied [25]. Six genes encode the vitellogenins of *C. elegans*. Genes *Cel-vit-1*-*5* are all located on the X chromosome but are not clustered [26]. *Cel-vit-6* is the only gene that is located on chromosome IV [26]. The six *C. elegans* vitellogenin genes encode four yolk polypeptides identified in oocytes and early embryos. *Cel-vit-1* and *2* encode YP170B; *Cel-vit-3*, *4* and *5* encode YP170A; and *Cel-vit-6* encodes the precursor of YP115 and YP88 [14], [27]. The precursor of YP115 and YP88 is proteolytically processed in the pseudocoelom of the worm [28], [29]. Recently, we performed an RNAi analysis and the results suggest that several convertases participate in this step, including a convertase which is expressed in the enterocytes [30].

*Oscheius tipulae*, which is analyzed in the present work, is a Rhabditid nematode found in the soil of São Paulo, Brazil [31], [32]. *Oscheius tipulae* is a widespread species with worldwide representatives, including the neotropical isolate CEW1 used in this study. The fact that hermaphroditic females are exclusively represented in laboratory cultures of the São Paulo isolate (males are extremely rare and difficult to maintain, unlike *C. elegans*), makes it easy to isolate and study the vitellins of *O. tipulae* [31]. Genetic, developmental and molecular data have been obtained from experiments in this species using the reference strain CEW1 [31], [33], [34].

Only two vitellogenin genes in *O. tipulae* have been characterized to date: *Oti-vit-6* [35] and *Oti-vit-1* (first described here). The *O. tipulae* genome is one of several nematode genomes being sequenced [36], but annotations have not been performed on the available contigs. *O. tipulae* vitellogenin genes encode three polypeptides: VT1 (175,2 kDa), VT2 (107 kDa) and VT3 (82 kDa) [31]. We previously demonstrated [35] that *Oti-vit-6* is homologous to *Ce-vit-6*, suggesting that this gene encodes the two minor *O. tipulae* vitellin polypeptides VT2 and VT3. Several molecular studies on the control of yolk protein gene transcription in *C. elegans* have been performed [5], [37], [38], [39].

This study describes the biochemical and molecular aspects of two vitellogenins (VT1 and OTI-VIT-6) of the nematode *Oscheius tipulae* and the purification and molecular characterization of the vitellins and their coding genes. We showed the position of the vitellins VT2 and VT3 in the precursor polypeptide (OTI-VIT-6) using Western Blotting with antisera raised against recombinant polypeptides of the N-terminal sequence of VT3 and C-terminal sequence of VT2. These recombinant polypeptides were also shown to interact with a polypeptide of unknown function previously identified in ligand-blotting experiments using native vitellins as probes [40]. The results of the phylogenetic analysis of the vitellogenins described in this study showed that these lipoproteins are orthologs of those from *Caenorhabditis*.

## Materials and Methods

### Animals

Monoxenic cultures of *O. tipulae* (strain CEW1) and *C. elegans* (strain N2) were maintained in NGM (Nematode Growth Medium) agar seeded with *Escherichia coli* NA22 as previously described [25]. Mass cultures were grown under vigorous aeration [41]. The worms were thoroughly washed in S buffer (50 mM potassium phosphate, 100 mM NaCl, pH 6.0), floated once in 30% (w/v) sucrose, incubated in S buffer at room temperature for 2 h for intestine cleansing, and washed once more before processing.

### Vitellin Purification

*O. tipulae* worms (CEW1) were obtained from asynchronous mass cultures using the *E. coli* strain NA22 as a food source and prepared as described above. The worms were subsequently washed two times in ultra-pure water through centrifugation at 1,000 rpm (200 *g*). The final worm slurry was slowly dropped into liquid nitrogen and stored at –70°C. All purification procedures were performed in the presence of protease inhibitors [1 mM Pefabloc (4-2-aminoethyl-benzenesulphonyl fluoride hydrochloride) - Roche™, 20 µM Pepstatin A – Sigma/Aldrich (São Paulo, Brazil) and 20 µM E-64– Sigma/Aldrich (São Paulo, Brazil)]. The frozen worm drops were ground to a powder under liquid nitrogen in a porcelain mortar using a porcelain pestle. The powder was thawed in homogenization buffer (40 mM Tris pH 7.4, 1.2 M NaCl, 2.0 mM CaCl_2_, 2.0 mM MnCl_2_), and the resulting homogenate was centrifuged at 1,500 *g* for 5 min. The supernatant was centrifuged at 10,000 *g* for 5 min, and the resulting supernatant was used for vitellin purification. Vitellin was purified using a ConA-Sepharose Column (GE Healthcare Bio-Sciences Corp., Piscataway, NJ, USA) [31]. The fractions were subjected to SDS-PAGE to identify samples enriched in the vitellins VT1, VT2 and VT3 [31]. The selected fractions were subsequently pooled, dialyzed at 4°C against 2 changes of 100 volumes of buffer A (10 mM Tris pH 8.0, 1.0 mM EDTA) in dialysis sacks (average flat width 25 mm; cutoff MW 12,000; Sigma-Aldrich, São Paulo, Brazil), and subjected to ion exchange chromatography using Resource Q columns (GE Healthcare Bio-Sciences Corp., Piscataway, NJ, USA) previously equilibrated with buffer A. The adsorbed polypeptides were subsequently eluted with a 0.1–1.0 M NaCl non-linear gradient in buffer A at a flow rate of 2.0 ml/min. The fractions containing VTG polypeptides were dialyzed, as described above, against PBS (phosphate buffered saline; 137 mM NaCl, 2.7 mM KCl, 10 mM Na_2_HPO_4_, 2 mM KH_2_PO_4_, pH 7.4) and concentrated using dry Sephadex G-75 resin (GE Healthcare Bio-Sciences Corp., Piscataway, NJ, USA) in a dialysis bag, as needed. The Sephadez G-75 dry beads were sprayed over the dialysis bag containing the protein solution. The swelling of the beads reduced the volume of protein solution inside the dialysis bag, increasing the protein concentration. The purified vitellins were stored at 4°C in the presence of protease inhibitors and used within 1 week of purification.

### N-terminal Microsequencing of O. tipulae vitellins

The chromatographically purified vitellogenin preparation was subjected to SDS-PAGE (10% gel). The gel was pre-run for 45 min at 10 mA without samples in Tris-glycine-glutathione buffer (25 mM Tris; 192 mM glycine; 10 mM glutathione; 0.1% (w/v) SDS, pH 8.3). The vitellogenin samples were dissolved in sample buffer containing β-mercaptoethanol [62.5 mM Tris; 2.5% (v/v) β-mercaptoethanol; 2.6% (w/v) SDS; 12.6% (v/v) glycerol; 0.5 mM EDTA; 0.5 mM EGTA; 0.01% (w/v) pyronin Y]. The fractionated polypeptides were subsequently electroblotted onto PVDF membranes (BioRad Trans-Blot Transfer Medium 0.2 µm) for 45 min at a constant current of 250 mA. Single bands corresponding to the vitellogenin polypeptides were excised and subjected to automatic protein sequencing using the automatic Edman degradation process, as previously described [42].

### Glycoprotein Detection

The glycoprotein detection was performed as previously described [43], with modifications. Briefly, *O. tipulae* vitellins were subjected to SDS-PAGE and blotted onto nitrocellulose membranes. The membranes were incubated with TBS (25 mM Tris pH 8.0, 137 mM NaCl, 15 mM KCl) containing 3% (w/v) oxidized bovine serum albumin (BSAox). BSAox was obtained by treating a BSA (Sigma-Aldrich, São Paulo, Brazil; acetone fraction V) solution with 10 mM periodic acid dissolved in 0.1 M sodium acetate, pH 4.5, for 6 h at room temperature in the dark. Subsequently, the BSAox was dialyzed twice against 200 volumes of TBS (25 mM Tris pH 8.0; 137 mM NaCl; 2.7 mM KCl) for 18 hours in dialysis sacks (average flat width 25 mm; cutoff MW 12,000; Sigma-Aldrich, São Paulo, Brazil). This procedure was used to remove the covalently linked carbohydrates from the BSA preparation. The BSAox-blocked membranes were subsequently incubated in a solution containing TBS-concanavalin A (50 µg/ml) and 4% BSAox (w/v) for 30 min at room temperature and washed with TBS. The membranes were then incubated with TBS containing 50 µg/ml HRP (horseradish peroxidase, Sigma-Aldrich) for 30 min at room temperature and finally washed with TBS. The bands were detected using development buffer [0.5 mg/ml 4-chloro-1-naphtol in 0.17% (v/v) methanol and 0.015% (v/v) H_2_O_2_ in TBS] and washed three times in water.

### RNA Purification

RNA extractions were performed by incubating the cleansed worms in Trizol:water (4∶1; v/v) for five min at room temperature followed by extractions using phenol:chloroform (1∶1; v/v) and chloroform. Total RNA was precipitated from the aqueous phase using isopropanol and incubated at –20°C in 70% (v/v) ethanol.

### VT1 cDNA Cloning and Sequencing

Dr. Marie-Anne Félix (Institute of Biology, École Normale Supérieur, IBENS) kindly provided plasmid pMA28, which contained a 5-kb insert isolated from an *O. tipulae* cDNA library. The plasmid DNA was digested with *Bam* HI, and a 2-kb restriction fragment was subsequently subcloned into the pTZ-19U (Fermentas UAB, Vilnius, Lituania) vector and sequenced using the appropriate primers. These sequences corresponded to the 5′ sequences of *vit-1* cDNA. We also sequenced other fragments of pMA28, but there were no matches with the *vit* sequences contained in GenBank, suggesting that pMA28 is a chimera of two different cDNAs cloned together.

The 5′ sequences were used as template for primer design and RT-PCR assays. A 2.5-kb fragment containing the 3′ sequences of the cDNA was amplified and cloned into pCR2.1 TOPO vector (Life Technologies, Carlsbad-CA, USA). The cDNA sequencing was performed with both plasmid and nested primers designed using previously deduced sequences.

### Selection of Expressed Regions

The C-terminal portion of the precursor protein OTI-VIT-6 corresponding to nucleotides 4,429 to 5,144 (GenBank Accession U35449) was excised from a bacteriophage clone isolated from a genomic library of *Oscheius tipulae* CEW1 [35]. The N-terminal portion of OTI-VIT-6 was obtained using RT-PCR with primers UPPER fw (^5′^CGCGGATCCGTTTCGGAACAATACT^3′^) and 11 rev (^5′^AGCACAAGACCTTCAACTG^3′^). The OTI-VIT-1 fragment (N-terminal sequence) was expressed from a fragment obtained using RT-PCR with primers EXP1_U_fw (^5′^GGATCCCTCCTTGAGCTCCCAATCCGTTT^3′^) and EXP2_L_rev (^5′^GTCGACAAACTGCTCGAAAGGCTTCTGCTC^3′^).

### Expression and Purification of Recombinant Polypeptides

The C-terminal region of OTI-VIT-6 was expressed from the fragment inserted into the pRSET C expression vector (Life Technologies). The 5′- fragment from *Oti-vit-6*, which contains no introns, was first cloned into the pCR 2.1 vector (Life Technologies, Carlsbad-CA, USA), and subsequently, a 996-bp region was excised using the restriction enzymes *Bam* HI and *Xho* I and subcloned into the pET 23a (+) expression vector (Merck KGaA, Darmstadt, Germany). The N-terminal region of OTI-VIT-1 was expressed from an amplified fragment subcloned into the pRSET C expression vector (Life Technologies Corp., Carlsbad-CA, USA). All recombinant plasmids were cloned into the BL21 Star DE3 (pLysS) *E. coli* strain (Life Technologies Corp., Carlsbad-CA, USA). Recombinant polypeptide expression was induced with 1 mM IPTG (isopropyl β-D-galactopyranoside) for 5 hours in SOB medium [2% (w/v) tryptone, 0.5% (w/v) yeast extract, 20 µM NaCl, 2.5 mM KCl, 10 mM MgCl_2_] containing 35 µg/ml chloramphenicol and 100 µg/ml ampicillin at 37°C with shaking at 200 rpm. The recombinant polypeptides were subsequently purified using a Pro Bond ™ Purification System (Life Technologies Corp., Carlsbad-CA, USA) with Ni^2+^ affinity columns according to the denaturing protocol in the kit instructions. The denaturants were removed and the purified polypeptides were solubilized using fractional dialysis against decreasing concentration of urea (6.0 M to 1.0 M) in PBS and finally against PBS alone. The samples were quantified using the BCA-assay (Thermo Fisher Scientific Inc., Rockford, IL USA) [44]. When the protein concentration was less than 0.1 mg/ml, the solution was transferred into a dialysis bag and subsequently covered with dry Sephadex G-75 resin (GE Healthcare Bio-Sciences Corp., Piscataway, NJ, USA) for 24 h at 4°C to obtain a 10 to 15X increase in the protein concentration of the solution. The affinity purified OTI-VIT-6 recombinant polypeptides were named P40 (N-terminal) and P26 (C-terminal) based on their estimated molecular masses in kDa. The OTI-VIT-1 recombinant polypeptide was named PVIT1, with an estimated molecular mass of 30 kDa.

### Anti-sera Production in Mice

These experiments were performed in strict accordance with the recommendations of the Brazilian National Council for the Control of Animal Experimentation (CONCEA). The Ethics Committee on the use of experimental animals (CEUA) of the Institute of Biomedical Sciences – University of São Paulo (Permit Number: 108; book 2, page 21) approved all protocols. All surgeries were performed using a mixture of the anesthetics ketamine and xylazine (10∶1), and suffering was minimized.

Immune sera against the purified recombinant polypeptides (P40, P26, and PVIT1) were produced in Balb/c mice. The animals were immunized subcutaneously with 5.0 µg of recombinant peptide in complete Freund's adjuvant as a primary immunization followed by two boost immunizations at intervals of four and two weeks, respectively. The boost doses contained the same quantity of protein emulsified in incomplete Freund's adjuvant. Blood was collected at one week after the last boost, and sera were obtained after clotting for 30 min at 37°C.

### Western Blotting

One volume of total protein extracts or solubilized recombinant polypeptides was mixed with one volume of 2X sample buffer [125 mM Tris; 5% (v/v) β-mercaptoethanol; 5.2% (w/v) SDS; 25.2% (v/v) glycerol; 1.0 mM EDTA; 1.0 mM EGTA; 0.03% (w/v) bromophenol blue] and boiled at 100°C for 10 min prior to loading and subsequently subjected to T = 10%, SDS-PAGE. The samples were transferred onto a nitrocellulose membrane in a Mini Protean II apparatus (Bio-Rad). The membranes were blocked for 2 h in TBS containing 5% (w/v) skimmed milk powder. The membranes were incubated overnight at 4°C with different dilutions of anti-sera in blocking solution. Following incubation with primary antibodies, the membranes were washed 3 times for 10 min each in TBS, incubated with 1∶2,000 peroxidase-conjugated anti-mouse IgG (Sigma/Aldrich, São Paulo SP, Brazil) diluted in TBS-milk for two hours at room temperature and washed 3 times for 10 min in TBS. The bands were detected using revelation buffer [0.5 mg/ml 4-chloro-1-naphtol in 0.17% (v/v) methanol and 0.015% (v/v) H_2_O_2_ in PBS] and washed three times with deionized water.

### Immunohistochemistry

Live worms were cut in half at the vulva on a polylysine-coated slide using a 15-blade iridectomy scalpel. A siliconized coverslip was placed over the worm sections without pressure. The slides were dipped in liquid nitrogen, and the coverslips were removed with a razor blade. The slides containing the frozen worm sections were fixed in methanol at –20°C for five min and subsequently dipped in acetone at the same temperature for five additional min. After fixation, the worms were rehydrated over a series of decreasing methanol concentrations from 95 to 30% (v/v) for two min incubation at each step. A final incubation was performed in PBS (phosphate buffered saline) for five min. The rehydrated material was blocked with 1% BSA (bovine serum albumin, fraction V)(w/v) and incubated with anti-serum at the appropriate dilution for 16 hours at 4°C. The slides were subsequently washed in PBS for ten min and incubated in the dark at 37°C for two hours with fluorescein-labeled anti-mouse IgG (Gibco BRL) diluted 1∶100. The slides were mounted with 10% glycerol (v/v) containing 0.5 mg/ml p-phenylenediamine and DAPI (4′,6-diamidino-2-phenylindole) (2 µg/mL). The photographs were captured with a Zeiss Axiophot DIC microscope equipped with a Hamamatsu C5985-02 camera (H. Photonics K.K., Hamamatsu, Japan) using the appropriate fluorescence filters and a 40x or 100x objective. A scale bar was added to each photo using a precision stage micrometer (0.01 mm; Bausch & Lomb, USA). The images were processed using Metamorph 3.0 (Universal Imaging Corp.) for final color and image superposition.

### Ligand Blotting Analysis

Whole worms were directly dissolved in sample buffer [62.5 mM Tris pH 6.8; 2,5% (v/v) β-mercaptoethanol; 2.6% (v/v) SDS; 12.6% (v/v) glycerol; 0.5 mM EDTA; 0.5 mM EGTA; 0.01% (w/v) pyronin Y] and boiling at 100°C for 10 min. Subsequently, the samples were then subjected to SDS-PAGE (T = 10%), and the proteins were electrophoretically transferred onto a nitrocellulose membrane (Hybond-C™, Amershan Biosciences) at 90 mA for 16 h at 4°C using a Mini Protean II apparatus (Bio-Rad) containing transfer buffer [20% (v/v) methanol; 195 mM glycine; 25 mM Tris pH 8.3]. The membranes were stained with Ponceau S and destained with TBS. The blot was incubated at room temperature for 2 h with TBS containing 5% (w/v) skimmed milk (TBS-milk). After buffer removal, the reaction with labeled recombinant polypeptides was incubated at 4°C in the dark with 20 or 50 µg of P40 or 40 or 100 µg of P26 in TBS-milk. The recombinant polypeptides were labeled with Fluorescein-5-EX-succinimidyl ester (Invitrogen/Molecular Probes, Carlsbad-CA, USA; cat. nr. F-6130) as previously described [40]. The following steps were performed at room temperature. The blot was washed three times (10 min each) in the dark with TBS and incubated for 2 h with TBS-milk containing anti-fluorescein peroxidase-conjugated antibodies (GE Healthcare Bio-Sciences Corp., Piscataway, NJ, USA) diluted 1∶1000 (v/v). After another three washes in TBS, the proteins were detected using the ECL Plus™ Western Blotting Detection System (GE Healthcare Bio-Sciences Corp., Piscataway, NJ, USA) and exposed to X-ray film at 25°C.

### Phylogenetic Analyses of the LLT (Large Lipid Transfer) Module

All nematode vitellogenin sequences available at the time of analysis (see Table S1) were aligned using Clustal X [45] and trimmed to obtain only the portion corresponding to the LLT module (Table S1) using JalView version 2 [46]. The phylogenetic and molecular evolutionary analyses were conducted using MEGA version 4 [47].

## Results

### Characterization of Oscheius tipulae Vitellogenins

**Figure 1:**
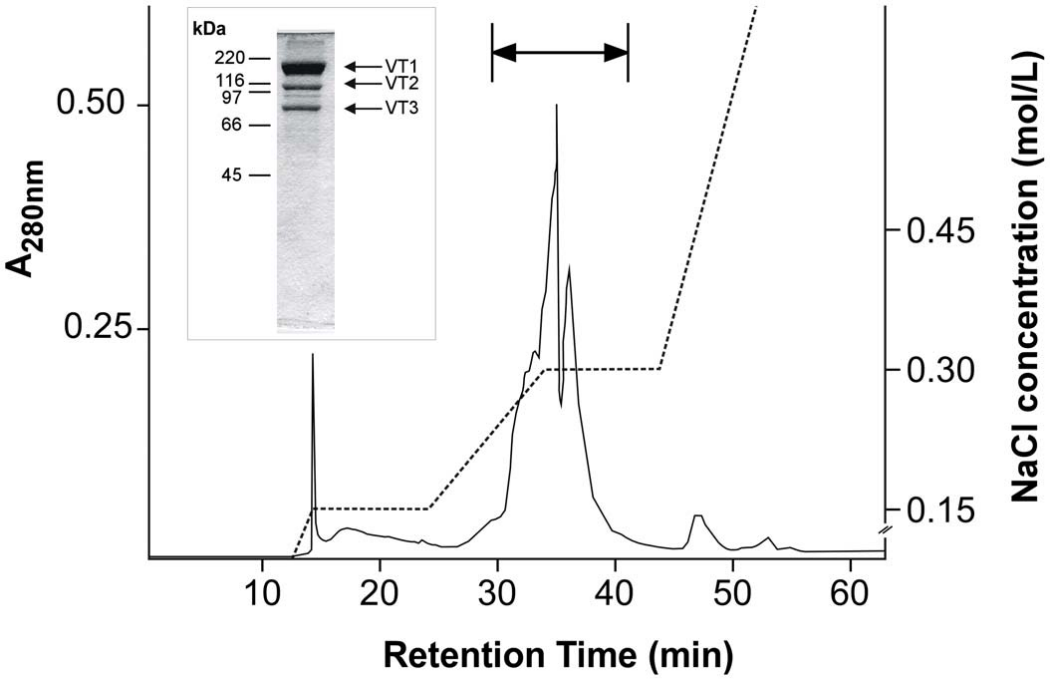
Vitellogenin purification using ion exchange chromatography. Con A-purified vitellogenin [31] was subjected to ion exchange chromatography in a MonoQ column (GE Healthcare Bio-Sciences Corp., Piscataway, NJ, USA). The absorbance of the eluate was continuously recorded at 280 nm (continuous line). The dotted line shows the discontinuous NaCl gradient. The inset at the left shows the Coomassie Blue R stained SDS-PAGE of the pooled fractions (arrow above the A_280nm_ profile).

The yolk proteins were purified using affinity chromatography over a ConA-Sepharose column followed by fractionation on an ion exchange column. This second step results in a >90% pure preparation of the three yolk polypeptides (VT1, VT2 and VT3) (**Figure 1**), as determined using SDS-PAGE, and is extremely sensitive to freezing and insoluble in PBS after freezing or lyophilization. We stored the yolk protein solutions at 4°C to maintain them in solution. Contamination with some intestinal proteases was not excluded in our preparations. Furthermore, lipid oxidation could occur at these conditions. To reduce the degradation of yolk proteins in these solutions, we added protease inhibitors and the antioxidant BHT [2,6-Bis(1,1-dimethylethyl)-4-methylphenol(butylated hydroxytoluene)] [48]; however, even under these conditions, the preparations were not stable for more than five days. An intrinsic proteolytic activity of the yolk proteins could also explain the instability of these preparations, as has previously been demonstrated in purifications of yolk proteins from other animals [49].

**Figure 2:**
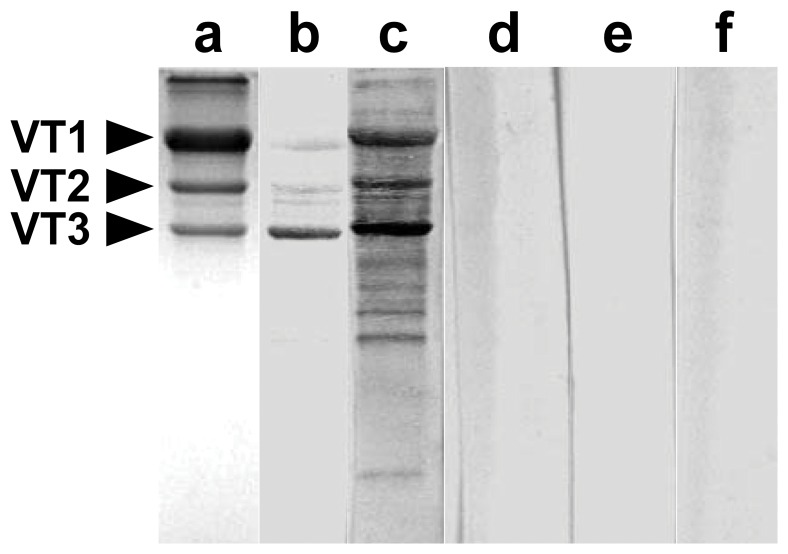
Concanavalin A (Con A) interaction with the purified vitellogenin polypeptides. Purified vitellins of *O. tipulae* were subjected to SDS-PAGE (T = 10%) and transferred to a nitrocellulose membrane (Hybond-C, Life Technologies). All lanes, except lane (**b**) with 0.5 µg of protein, contained 5 µg of purified *O. tipulae* vitellogenin. Each lane of the membrane was cut and incubated separately in the presence (**b**, **c**, **d** and **f**) or absence (**e**) of Concanavalin A (50 µg/ml). (**a**) Coomassie Blue-stained SDS-PAGE of purified vitellins. (**b** and **c**) Membrane strips incubated with horseradish peroxidase (50 µg/mL). (**d**) Membrane strip incubated in the absence of horseradish peroxidase. (**f**) Membrane strip incubated in the presence of horseradish peroxidase (50 µg/mL) and 0.2 M α-methyl mannoside.

To determine whether the *O. tipulae* polypeptides contain sugar residues, we employ an extremely sensitive blotting protocol to detect small amounts of glycoproteins using Concanavalin A. **Figure 2** shows that all of the polypeptides are linked to mannose/glucose residues. The intensity of the band corresponding to VT3 suggests that this polypeptide has a higher molar ratio of mannose/glucose than the other vitellin polypeptides.

### Isolation and Characterization of a New Vitellogenin Gene from O. tipulae

We analyzed *O. tipulae* cDNA clone pMA28 and obtained a partial sequence from a gene with high similarity to other nematode vitellogenins. The mRNA was amplified in successive RT-PCR steps and partially sequenced (see Materials and Methods).

**Figure 3:**
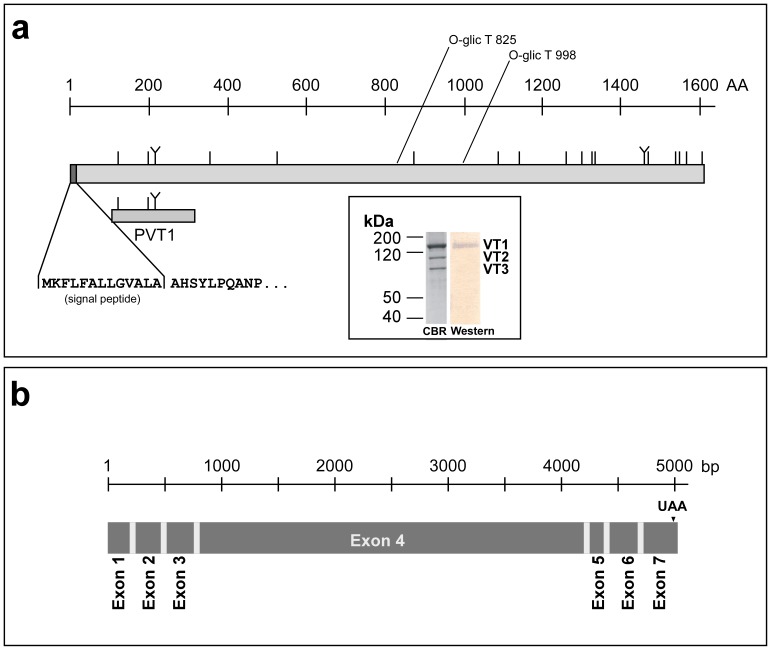
Graphical representation of the gene *Oti-vit-1* and vitellogenin OTI-VIT-1 and immunodetection of VT1. (**a**) Graphical representation of the conceptual protein, OTI-VIT-1. The vertical lines show the position of each cysteine in the molecules; the “Y”-shaped lines show the position of the “CXXC” motif. The position of the recombinant polypeptide PVT1, expressed in *E. coli*, and the position of the signal peptide obtained after N-terminal sequencing of the vitellin polypeptide VT1 are indicated beneath the protein representation. The inset shows a Coomassie Blue stained SDS-PAGE of 5 µg of purified *O. tipulae* vitellins (CBR) and the corresponding Western Blot (Western) of the same sample. The vitellins were separated using SDS-PAGE (T = 10%) and the Western Blot analysis was performed with mouse anti-PVT1 serum. (**b**) Graphical representation of the *Oti-vit-6* gene. Using the cDNAs obtained with RT-PCR, we annotated the *Oti-vit-1* sequence present in contig 4918 of the ongoing genome sequencing of *O. tipulae* at the Blaxter lab [36] (see Figure S1 for more details).

A recombinant polypeptide (PVT1) corresponding to the N-terminal region of the *Oti-vit-1* clone was used to raise anti-serum in mice. This anti-serum recognized VT1 in a Western blot of whole adult hermaphrodite worms (**Figure 3A**, insert), which suggests that this gene encodes VT1. The same analysis yielded negative results for immature *O. tipulae* larvae (not shown). In addition, the N terminus of VT1was microsequenced [AHSYLE] and the results showed that the sequence was identical to the conceptually translated sequence from the cloned cDNA (GenBank Accession JX081582) minus the putative signal peptide, clearly indicating that *Oti-vit-1* encodes VT1. One of the contigs obtained during the sequencing of the *O. tipulae* genome [36] contains the entire *Oti-vit-1* sequence (see **Figure S1**). We were able to map all of the cDNA sequences to *Oti-vit-1* and found that this gene contains at least six small introns (**Figure 3B** and **Figure S1**), as is typical in *O. tipulae* genes [34], [35]. The predicted protein encoded by *Oti-vit-1* has a theoretical molecular mass of 187,023 Da, which is similar to the mass determined for VT1 using SDS-PAGE [31]. These results strongly show that *Oti-vit-1* encodes the vitellin polypeptide VT1 (**Figure 3B**).

### Proteolytic Processing of OTI-VIT-6 Precursor Protein into VT2 and VT3

OTI-VIT-6 is a large precursor protein (1660 amino acids; predicted molecular mass of 192,000 kDa) that is potentially proteolytically processed to generate VT2 and VT3, as occurs with its homolog CEL-VIT-6 [31], [35]. To map the position of VT2 and VT3 in the precursor vitellogenin protein OTI-VIT-6, we expressed several portions of the precursor in *E. coli*. The recombinant polypeptides presented different solubility properties, indicating that OTI-VIT-6 have a 3D arrangement similar to that previously suggested for other large lipid transfer proteins (LLTPs) [50].

**Figure 4:**
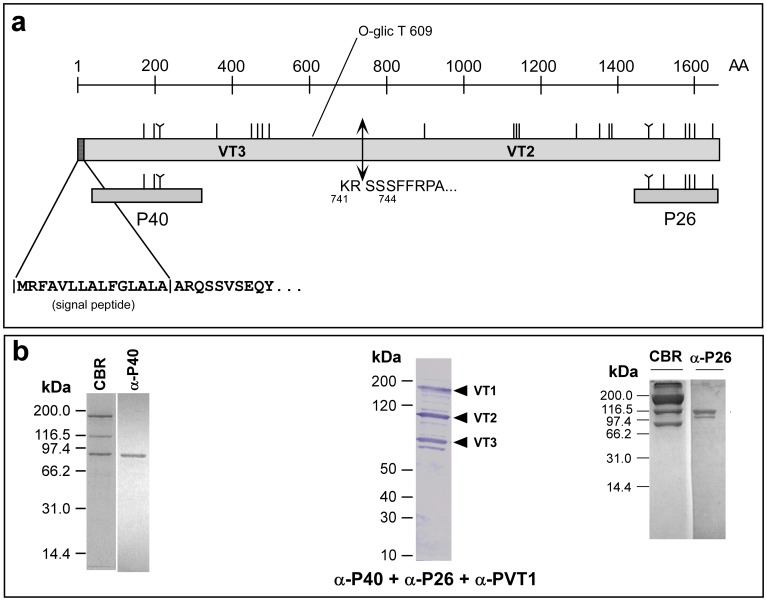
Positioning yolk polypeptides VT2 and VT3 in the vitellogenin precursor OTI-VIT-6. (**a**) Graphical representation of OTI-VIT-6. The vertical lines show the position of each cysteine in the molecules; the “Y”-shaped lines show the position of the “CXXC” motif. The positions of the recombinant polypeptides expressed in *E. coli* are shown underneath the protein representation. The cleavage sites of the signal peptide and processing of the precursor were obtained using N-terminal sequencing of the mature VT2 and VT3. (**b**) Coomassie Blue-stained SDS-PAGE of purified *O. tipulae* vitellins (CBR) and the immunoblots using mouse anti-P40 (α-P40) and anti-P26 (α-P26) sera. The results from the analysis with all three anti-sera (α-P40+ α-P26+ α-PVT1) in the same blot are also shown. Each of the Western blotting strips represent a different SDS-PAGE experiment.

We expressed the C-terminal portion of OTI-VIT-6 in *E. coli*. The recombinant polypeptide (P26) is 279 amino acids in length with a predicted mass of 26 kDa and considering its origins as part of a lipid transport protein, this region presents several unusual properties. P26 is highly soluble, which suggests that this region of the protein is not involved in lipid interaction. X-ray and NMR studies have shown that the region of lamprey lipovitellin [52] that is homologous to P26 does not fold as a stable portion of the protein but rather is mobile and most likely does not interact with lipids. P26 also shows high aggregative potential during SDS-PAGE, which is likely due to the high proportion of cysteine residues present in this region of the polypeptide (see **Figure 4**).

Two polypeptides from the N-terminal region of OTI-VIT-6 were also expressed in *E. coli*. One of the polypeptides was insoluble in the absence of SDS (not shown). The other polypeptide, P40, was partially soluble in the absence of detergents and corresponded to the N-terminal region of the precursor protein without the signal peptide. Similar results were obtained with the recombinant polypeptides from portions of the lamprey lipovitellin, which are insoluble in the absence of detergents or chaotropic agents [50].

The N-terminal sequences obtained from SDS-PAGE-purified VT2 [(S)IIFRX(A)] and VT3 [ARQ] were compared with the predicted sequence of OTI-VIT-6. Although the N-terminal sequence obtained from VT3 is short, the three PTH-amino acid peaks obtained on the ion exchange chromatogram were sharp and high with no other contaminants. These results allowed us to to determine the signal peptide cleavage site (^14^LA|AR^17^) and infer that both yolk polypeptides are located in the precursor protein. Following proteolytic processing, the precursor yields VT2 and VT3 (**Figure 4**).

The recombinant polypeptides P26 and P40 were used to raise antibodies in Balb/c mice and the sera was applied in Western Blotting experiments of purified vitellogenin preparations and whole worm extracts (**Figure 4**). These results show that anti-P26 specifically recognizes VT2 and confirms that VT2 corresponds to the C-terminal portion of OTI-VIT-6. By exclusion, we also concluded that VT3 was located in the N-terminal portion of OTI-VIT-6, as previously suggested by the N-terminal sequence of this yolk polypeptide, and confirmed by the use of anti-P40 serum (**Figure 4**).

*C. elegans* vitellogenins were not detected with any of the antisera raised against *O. tipulae* recombinant polypeptides P26 and P40 (results not shown), indicating that VIT-6 cleavage products of *O. tipulae* and *C. elegans*, although homologous [31], [35], clearly differ in their immunological properties.

### Vitellogenin Uptake and Storage in O. tipulae Oocytes

**Figure 5:**
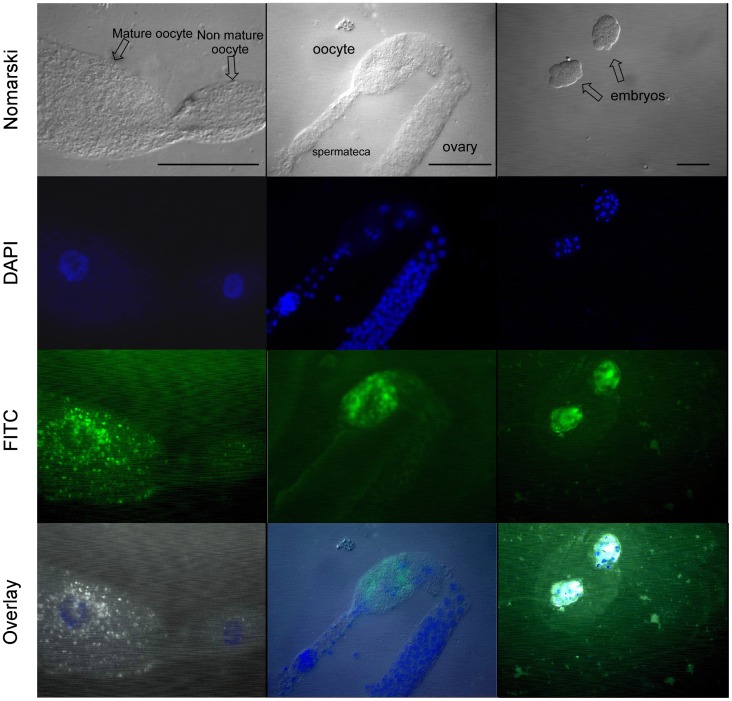
Immunofluorescence detection of yolk granules in the *O. tipulae* ovary and embryos using anti-P26 sera. Worms were cut open with iridectomy blade over a gelatin-subbed slide, fixed and permeabilized. The slides were incubated with anti-P26 serum diluted to 1∶100 and subsequently incubated with FITC anti-mouse IgG in the same dilution. The slides were visualized using an Axiophot Zeiss photomicroscope, and photos were captured with epifluorescence (DAPI and FITC filters) or DIC for each field. The black scale lines in the first row (DIC/Nomarski photos) correspond to 50 µm.

Using anti-P26 serum in an immunofluorescence assay, we were able to detect clear signals in mature, but not in immature, oocytes (**Figure 5**). The fluorescence signal was also detected in embryos with a small number of cells. No signal was detected in the intestine or pharynx of young adults (results not shown), although vitellogenin is synthesized in the intestine [31]; this result suggests that vitellogenin is not stored in the intestine before being secreted into the pseudocoelom, as demonstrated for *C. elegans* [28]. Our data are consistent with data from *C. elegans*, which suggests that *O. tipulae* VTG is continuously synthesized, secreted, processed and taken up by growing oocytes.

### The VTG Interaction with a Single 100 kDa Band was Shown Using Ligand Blotting with Recombinant Polypeptides

**Figure 6:**
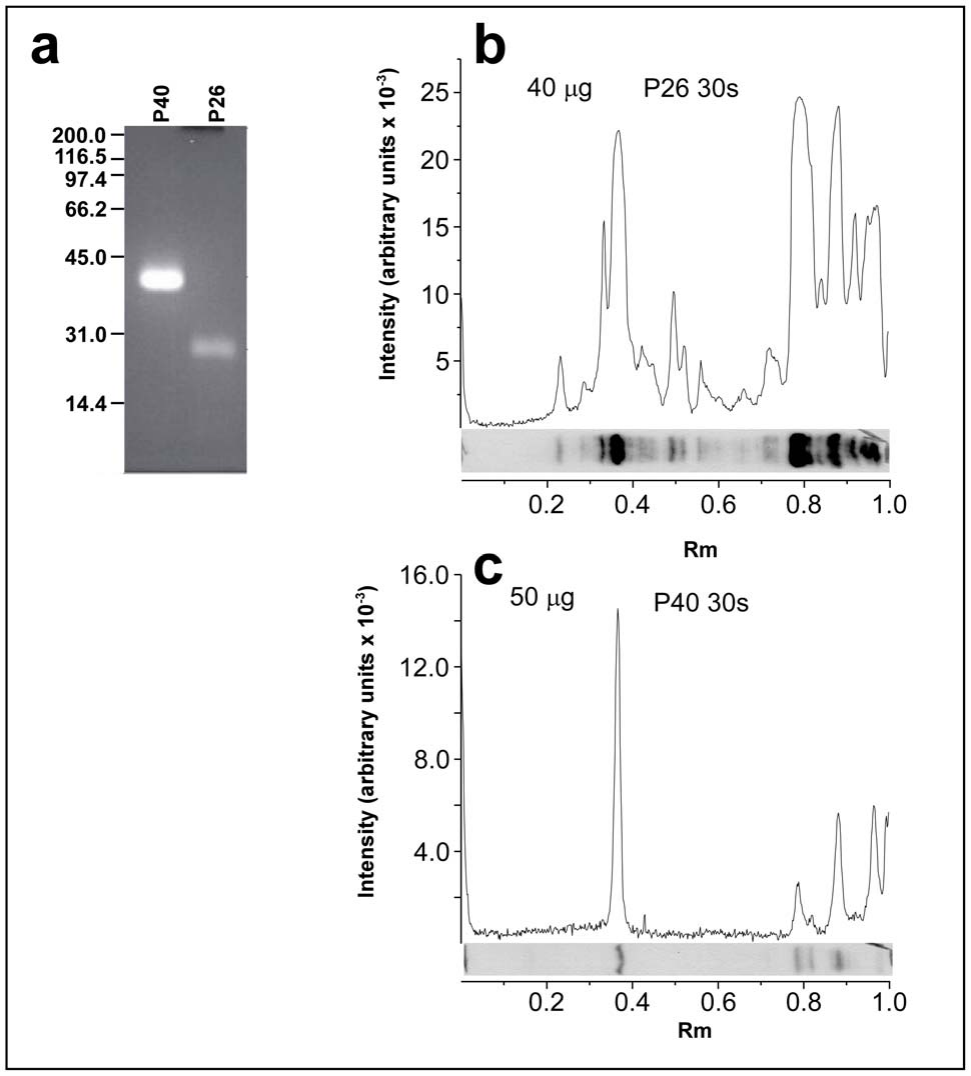
Ligand-blotting analysis of the interaction between recombinant polypeptides P40 and P26 with proteins from whole worm extracts. Forty µg of recombinant P40 and P26 were labeled with fluorescein, subjected to SDS-PAGE and photographed under a short-UV lamp (a). Whole worm extract was subjected to SDS-PAGE (T = 10%) and transferred onto Hybond C membranes. Membrane strips containing the fractionated extract were incubated with fluorescein-labeled P40 (50 µg; 1125 pmoles)(b) and P26 (40 µg; 1520 pmoles) (c). After extensive washing, the bound recombinant fluorescein-labeled polypeptides were detected using an anti-fluorescein antibody labeled with peroxidase. The peroxidase activity was detected using chemiluminescence. The chemiluminescence reaction was exposed to Hyperfilm ECL (GE Healthcare Bio-Sciences Corp., Piscataway, NJ, USA) for 30 s in both experiments.

Previous results [40] have shown that *O. tipulae*-purified vitellin preparations interact with a polypeptide of 100 kDa (P100) present in worm extracts fractionated using SDS-PAGE. This interaction is stage and sex-specific, and occurs only when the extracts of adult hermaphroditic worms are used. To determine whether this protein-protein interaction depends on carbohydrates or lipids associated with the native vitellins, we performed similar experiments using fluorescein-labeled recombinant polypeptides P40 and P26 (described above). As shown in our previous work [40], P100 is the primary signal observed in this assay. Due to the greater number of lysines in the P40 sequence compared with that of P26, P40 was more efficiently labeled using the fluorescein succinimidyl ester (Figure 6A). Using approximately the same molar amount of both polypeptides in the ligand-blotting assay, a stronger signal was present in the P100 region when P26 was used than when P40 was used. Nevertheless, the binding of P40 is more specific when we normalize the data using the unspecific binding portions of the blot (between Rm 0.8 and 1.0; Figure 6B and 6C) that correspond to the anti-fluorescein serum interaction with unlabeled components of the worm extract [40]. This nonspecific region shows lower fluorescence signal in the P40 lane than in the P26 lane.

### Phylogenetic Analysis of the N-terminal Region of Nematode Vitellogenins

**Figure 7:**
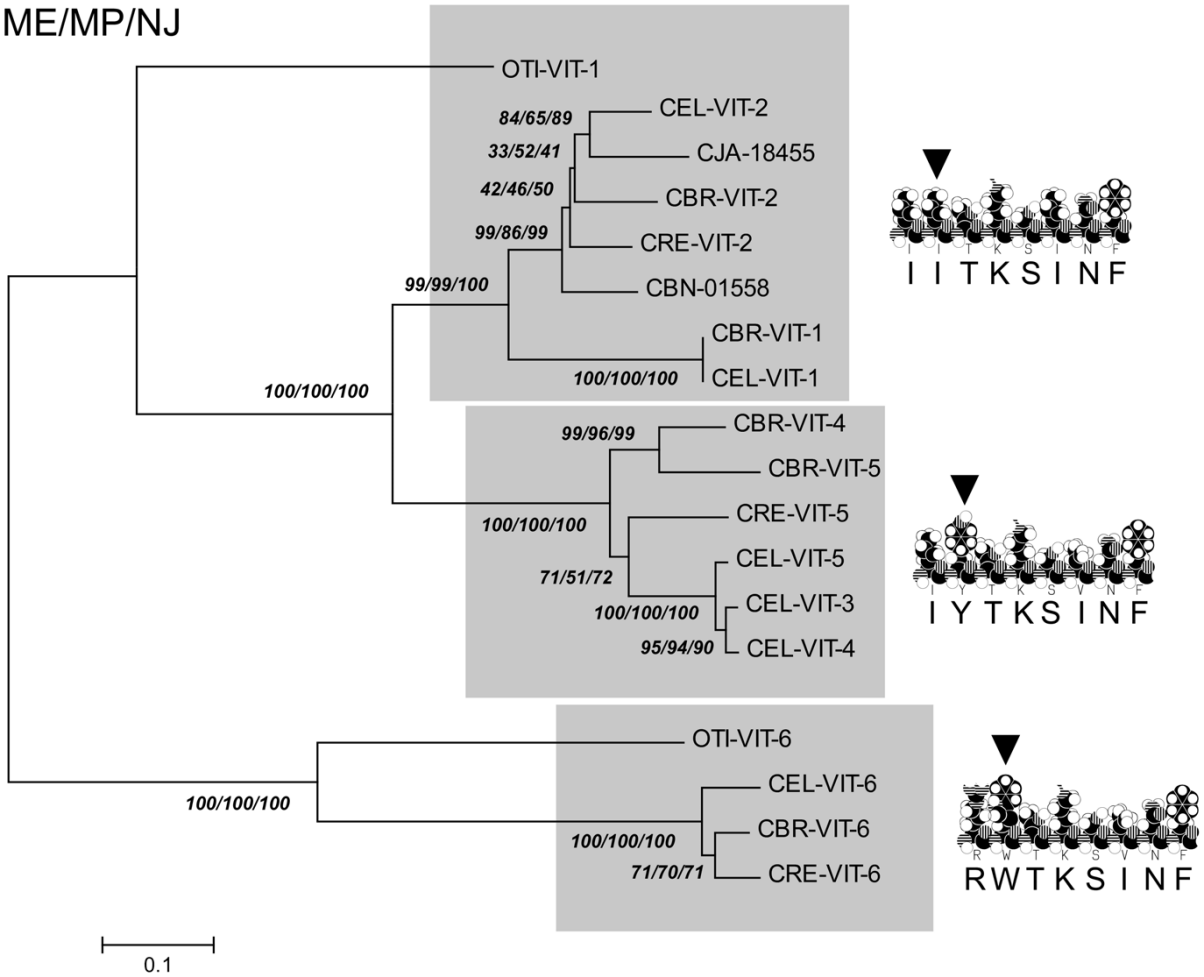
Phylogenetic analysis of the LLT module of a nematode vitellogenin amino acid sequence. The LLT module of all fifteen yolk protein sequences known from *Caenorhabditis* spp. and *Oscheius tipulae* were aligned with Clustal X, using the Blosum64 score matrix and the default values of the software for gap penalties. The alignment was subjected to phylogenetic analysis using Minimum Evolution (ME), Maximum Parsimony (MP) and Neighbor-Joining (NJ) methods. The numbers shown in the unrooted tree represent the bootstrap values obtained from 10,000 replicate trees (ME, MP and NJ). The shadowed branches correspond to the amino acid sequences containing the consensus VTGR binding site obtained from **Table S2** shown at right of the tree. The bar at the lower left shows the number of substitutions per 100 amino acid residues.

To further analyze the N-terminal region of nematode vitellogenins, we have aligned the amino acid sequences of the LLT module [6] of all annotated vitellogenins from several *Caenorhabditis* species and *O. tipulae* (Table S1). Previous results obtained in fish vitellogenin suggest that the binding site of different vitellogenins to their receptor is conserved in several oviparous animals (vertebrates and invertebrates) and is localized inside the LLT module [6], [53]. Based on the results of [53], we were able to infer the putative binding site of all nematode vitellogenins. Analyzing the VTGR binding motifs of all known nematode vitellogenins, we grouped the polypeptides into two different families according to the properties of the amino acids present at position 2 of the motif (Table S2). One group contains either isoleucine or valine (branched residues), and the other group has either tryptophan or tyrosine (aromatic residues) in this position. By applying three different tree construction methods to the LLT module alignment, we constructed a dendrogram of all of the amino acid sequences (Figure 7). As expected from previous work [5], [54], the polypeptide homologs YP170A and YP170B (including *O. tipulae* VT1) have a single ancestor and form two distinct clades. VIT-6 related proteins, in contrast, form a distinct group. The dendrogram illustrates that the putative binding sites that contain aromatic residues in position 2 comprise proteins that are present in the VIT-6 and YP170A clades, which are distantly related.

## Discussion

### Sequencing of O. tipulae Vitellogenin Genes and Peptides

The N-terminal sequencing of VT1 resulted in clear non-ambiguous signals that corresponded to the amino acid sequence Ala-His-Ser-Tyr-Leu-Asp (AHSYLD), which suggests that this region of the SDS-PAGE contains a single polypeptide. These data also suggest that VT1 of *O. tipulae* is different from its homolog, YP170, which is the primary yolk polypeptide in *C. elegans* and is composed of two different polypeptides with almost the same molecular mass (YP170A and YP170B) [5]. Nevertheless, VT1 could contain two polypeptides that are encoded by recently duplicated genes coding for identical N-terminal sequences. The results of the non-denaturing PAGE showed only one native yolk protein band [31], although two bands were expected from previous studies with *C. elegans* [51]. This intriguing result in *O. tipulae* could be explained by the presence of only one YP170 polypeptide homolog, which represents the heterotrimeric and dimeric complexes that have previously been detected in *C. elegans* [51]. Another possibility is that only one of the VT1 polypeptides is glycosylated and the other was lost during the ConA-Sepharose chromatography purification step.

Using this same approach, VT2 and VT3 N-terminal sequences were also determined using automatic Edman degradation. The exact cleavage position from the position of the precursor (OTI-VIT-6) occurs after a pair of basic amino acids (^741^LysArg|SerSer^744^). This same pair of basic amino acids and the P1, P2, P3, P4, P5 and P6 amino acids were also observed in CEL-VIT-6, and it has been suggested that these polypeptides were most likely cleaved by a furin-like convertase. We previously suggested [5] that this processing step in *C. elegans* is performed by KPC-1, but recent results from our lab [30] suggest that other members of these worm convertases could be involved. Functional redundancy among convertases has previously been described for mammalian furins [55], [56].

Thompson and Banaszak [52] published a three-dimensional model of lamprey (*Ichthyomtzon unicuspis*) lipovitellin. Results of the computer modeling (not shown) performed using OTI-VIT-6 and OTI-VIT-1 amino acid sequences allowed us to identify domains present in both vitellins and other members of the LLTP family [6], [50]. One example of conserved amino acid sequences identified in both *O. tipulae* vitellins and other lipoproteins is the N-terminal and C-terminal CXXC motifs. These residues are also found in VTG-related proteins, such as the von Willebrand factor and apolipoprotein B [57]. In prokaryotes, such as *E. coli*, this motif is characteristic of thioredoxin isomerases, and PDIs (Protein-Disulfide-Isomerases) [58]. PDIs are also present in eukaryotes and humans and are one of the subunits of MTP (Microsomal Triglyceride Transfer Protein), which is a lipid-transfer protein that plays a central role in lipid metabolism and obesity disorders [59]. Although the function of the CXXC motif in worm VTGs is unknown, it is reasonable to consider a certain degree of isomerase function for these lipoproteins.

### Interactions of Recombinant Polypeptides

We have previously shown that chromatographically purified *O. tipulae* vitellogenin strongly interacts with a polypeptide, P100, in a species-, sex- and stage-specific manner [40]. We were able to show that the recombinant P40 can also interact preferentially with P100, when compared to P26, which corresponds to the C-terminal portion of OTI-VIT-6.

Because the vitellogenin of vertebrates interacts with its receptor through a motif present in the N-terminal region of the protein [58], [59], it is tempting to speculate that P100 is the vitellogenin receptor of *O. tipulae*. Another explanation is that *O. tipulae* vitellogenins interact with the microsomal triglyceride transfer protein (MTP), which was previously identified in *C. elegans* [60] and is involved in vitellogenin assembly in *Xenopus laevis* [61]. Using RNAi experiments, we have previously shown that the knockdown of the MTP gene of *C. elegans* (*dsc-4*) affects vitellogenin transport in the intestine [62]. The MTP of *C. elegans* is exactly the same size as P100, but attempts to isolate and characterize P100 using pull-down experiments were unfruitful [62].

### Phylogenetic Analysis of Nematode Vitellogenin Polypeptides

The phylogenetic tree constructed with the nematode LLT modules is robust and congruent with trees constructed using whole vitellogenin amino acid sequences [35]. The vitellogenins of nematodes form two different families. The more distant proteins are proteolytically processed and encoded by *vit-6* genes. The putative receptor binding motifs of those vitellogenins do not have the same distribution in the tree when position two of the consensus is considered (Figure 7). Amino acids with similar chemical properties in the binding site could indicate either a phylogenetic or a functional relationship. Thus, the second hypothesis is probable in this case. This functional relationship seems even more likely when we consider that in *C. elegans*, the polypeptides grouped according to the chemical characteristic of their putative binding sites (YP170A, YP115 and YP88) form a single protein complex [51]. In contrast, polypeptides belonging to the YP170B clade (that forms a dimer in *C. elegans* [51]) present a branched residue at position 2.

Based on the phylogenetic and structural results presented here, we propose that the two protein complexes formed by the Rhabditid VTG subunits [51] are functionally different. The results obtained by other groups suggest that Rhabditid vitellogenin polypeptides are involved in worm survival [63] and are differently modified in aging worms [64]. Those polypeptides most likely have different physiological functions in the worm homeostasis.

## Supporting Information

Figure S1 **Gene** ***Oti-vit-1*** **and its partial cDNAs.** The complete sequence of *Oti-vit-1* was identified in *contig_4918* (11,093 bases) from the *Oscheius tipulae* genome project (Available: http://nematodes.org/downloads/959nematodegenomes/blast/db/Oscheius_tipulae_clc3_1.fna. Accessed 2012 Dec 4). The gene is 5,072 bp long and is located from position 2,424 to 7,495 (ATG to TAA) in the contig sequence. The figure shows the position of the three partial cDNAs obtained as described in Materials and Methods. The arrowheads under the 5′ portion of the cDNA show the position of oligonucleotides EXP1_U_fw and EXP2_L_rev used to amplify the cDNA fragment for the expression of PVT1. The GenBank accession numbers are provided under each of the partial cDNAs.
(TIF)

Table S1 **Vitellogenin sequences used for phylogenetic analysis.**
(DOCX)

Table S2 **VTG-VTGR putative binding sites on the nematode vitellogenins.**
(DOCX)
