# Supplementary figures and images for: The Molecular and Structural Characterization of Two Vitellogenins from the Free-Living Nematode Oscheius tipulae
Source: PLoS One. 2013 Jan 7;8(1):e53460. doi: 10.1371/journal.pone.0053460 (PMC3538542; doi:10.1371/journal.pone.0053460)

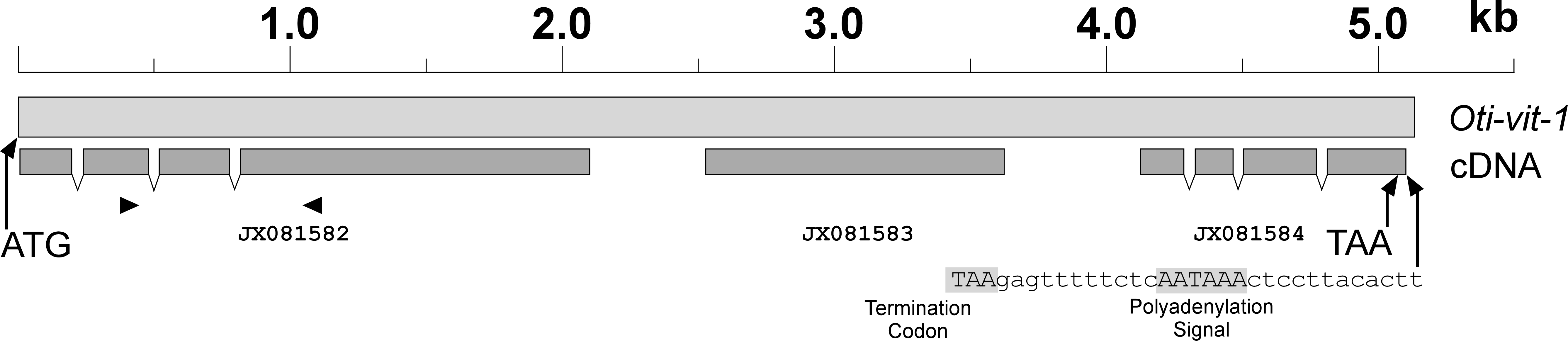

Supplement: Figure S1 — Gene Oti-vit-1 and its partial cDNAs. The complete sequence of Oti-vit-1 was identified in contig_4918 (11,093 bases) from the Oscheius tipulae genome project (Available: http://nematodes.org/downloads/959nematodegenomes/blast/db/Oscheius_tipulae_clc3_1.fna. Accessed 2012 Dec 4). The gene is 5,072 bp long and is located from position 2,424 to 7,495 (ATG to TAA) in the contig sequence. The figure shows the position of the three partial cDNAs obtained as described in Materials and Methods. The arrowheads under the 5′ portion of the cDNA show the position of oligonucleotides EXP1_U_fw and EXP2_L_rev used to amplify the cDNA fragment for the expression of PVT1. The GenBank accession numbers are provided under each of the partial cDNAs. (TIF) [file pone.0053460.s001.tif]
