# Supplementary material for: The Molecular and Structural Characterization of Two Vitellogenins from the Free-Living Nematode Oscheius tipulae
Source: PLoS One. 2013 Jan 7;8(1):e53460. doi: 10.1371/journal.pone.0053460 (PMC3538542; doi:10.1371/journal.pone.0053460)
Supplement: Table S1 — Vitellogenin sequences used for phylogenetic analysis. (DOCX) [file pone.0053460.s002.docx]

**Table S1**

Vitellogenin sequences used for phylogenetic analysis

| **Gene name** | **Vitellogenin** | **Accession nr^§^** | **LLT module position in the protein** | **Species** |
| --- | --- | --- | --- | --- |
| CBN01558 | YP170B | CN07961* | 1-419 | *C. brenneri* |
| *Cbr-vit-1* | YP170B | A8XPS2 | 1-418 | *C. briggsae* |
| *Cbr-vit-2* | YP170B | A8XKA3 | 1-421 | *C. briggsae* |
| *Cbr-vit-4* | YP170A | A8XJI2 | 1-416 | *C. briggsae* |
| *Cbr-vit-5* | YP170A | A8XJK4 | 1-423 | *C. briggsae* |
| *Cbr-vit-6* | YP88+115 | A8WRB9 | 1-441 | *C. briggsae* |
| *Cel-vit-1* | YP170B | P55155 | 1-417 | *C. elegans* |
| *Cel-vit-2* | YP170B | P05690 | 1-418 | *C. elegans* |
| *Cel-vit-3* | YP170A | Q9N4J2 | 1-418 | *C. elegans* |
| *Cel-vit-4* | YP170A | P18947 | 1-418 | *C. elegans* |
| *Cel-vit-5* | YP170A | P06125 | 1-418 | *C. elegans* |
| *Cel-vit-6* | YP88+115 | P18948^₪^ | 1-443 | *C. elegans* |
| CJA18455 | YP170B | JA29771* | 1-421 | *C. japonica* |
| *Cre-vit-2* | YP170B | E3LE68 | 1-420 | *C. remanei* |
| *Cre-vit-5* | YP170A | E3LCE2 | 1-423 | *C. remanei* |
| *Cre-vit-6* | YP88+115 | E3M6R6 | 1-442 | *C. remanei* |
| *Oti-vit-1* | VT1 | JX081582** JX081583 JX081584 | 1-404 | *O. tipulae* |
| *Oti-vit-6* | VT2+3 | AAB49749 | 1-419 | *O. tipulae* |

**^§^** All accession numbers, except those with asterisks, were obtained from UniProtKB (http://www.uniprot.org/help/uniprotkb)

^₪^ Isoform c at UniProtKB

* Accession numbers obtained from the Wormbase (http://www.wormbase.org/)

**Translated from partial cDNA sequences (accession numbers were obtained from GenBank)
