# Supplementary material for: The Molecular and Structural Characterization of Two Vitellogenins from the Free-Living Nematode Oscheius tipulae
Source: PLoS One. 2013 Jan 7;8(1):e53460. doi: 10.1371/journal.pone.0053460 (PMC3538542; doi:10.1371/journal.pone.0053460)
Supplement: Table S2 — VTG-VTGR putative binding sites on the nematode vitellogenins. (DOCX) [file pone.0053460.s003.docx]

**Table S2**

VTG-VTGR putative binding sites* on the nematode vitellogenins

| **YP170B** | | **YP170A** | | **YP115–YP88** | |
| --- | --- | --- | --- | --- | --- |
| **Genes** | **Site** | **Genes** | **Site** | **Genes** | **Site** |
| *Cbr-vit-2* | IITKSVNF | *Cel-vit-3* | IYTKSVNF | *Cel-vit-6* | RWAKSINF |
| *Cel-vit-2* | IITKSINF | *Cel-vit-4* | IYTKSVNF | *Cbr-vit-6* | RWAKSINF |
| *Cja_18455* | IITKSINF | *Cbr-vit-4* | IYTKSVNF | *Cre-vit-6* | RWAKSINF |
| *Cre-vit-2* | IITKSINF | *Cel-vit-5* | IYTKSVNF | *Oti-vit-6* | QWTKSINF |
| *Oti-vit-1* | LVTKSTNF | *Cbr-vit-5* | IYTKSVNF | **Consensus** | **RWTKSVNF** |
| *Cbn_01558* | IITKSINF | *Cre-vit-5* | IYTKSVNF |  |  |
| *Cbr-vit-1* | IVTKSVNF | **Consensus** | **IYTKSVNF** |  |  |
| *Cel-vit-1* | IVTKSVNF |  |  |  |  |
| **Consensus** | **IITKSINF** |  |  |  |  |
|  |  |  |  |  |  |
|  |  |  |  |  |  |

* - Results obtained according to [55].
